# Supplementary material for: Modulating FOXO3 transcriptional activity by small, DBD-binding molecules
Source: eLife. 2019 Dec 4;8:e48876. doi: 10.7554/eLife.48876 (PMC6919977; doi:10.7554/eLife.48876)
Supplement: Supplementary file 1. [file elife-48876-supp1.pdf]

Synthesis of S9 and S9ox

Synthesis of S9 and S9ox 1
General 1
Reaction scheme 1
Preparation of substrates and corresponding NMR spectra 2
Preparation of oxalate salt derived from S9 and corresponding NMR spectra 9

General

Chemicals and solvents were either purchased from commercial suppliers. For thin-layer chromatography (TLC), silica gel plates Merck 60 F254 were used and compounds were visualized by irradiation with UV light and/or by treatment with a solution ninhydrine followed by heating. Column chromatography was performed using silica gel Merck 60 (particle size 0.063-0.200 mm). ^{1}H-NMR, ^{13}C-NMR spectra were recorded on Bruker AVANCE III 400. Chemical shifts for protons are given in δ relative to tetramethylsilane (TMS) and are referenced to residual protium in the NMR solvent (DMSO-d_6: δ = 2.50 ppm, Methanol-d_4: δ = 4.87 ppm). Chemical shifts for carbon are referenced to the carbon in NMR solvent (DMSO-d_6: δ = 39.52 ppm,, Methanol-d_4: δ = 49.00 ppm. The coupling constants J are given in Hz. IR DRIFT spectras were recorded with Nicolet AVATAR 370 FT-IR in cm^{-1}. High-resolution mass spectras were recorded with LCQ Fleet spectrometer.

Reaction scheme

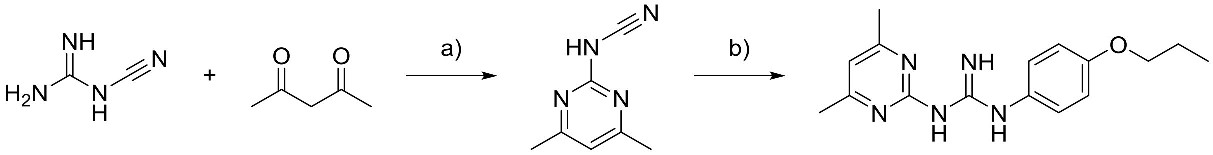

Scheme 1: Synthetic route to S9. Reagents and conditions: a) NaOH, H_2O, reflux, 22% yield, b) 4-propoxyaniline, EtOH, reflux, 47% yield.

Preparation of substrates

N-(4,6-Dimethylpyrimidin-2-yl)cyanamide

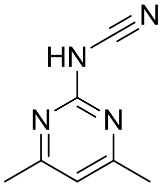

N-(4,6-Dimethylpyrimidin-2-yl)cyanamide was prepared according to previously reported procedure.[1]

Cyanoguanidine (5.0 g, 60 mmol), acetylacetone (9.0 g, 90 mmol) were added to a solution of NaOH (0.3M, 40 ml) and the reaction mixture was stirred under reflux 48 h. Then the mixture was cooled to 4 °C, solids were filtered and washed with minimal amount of water. Filtrate cake was recrystallized from boiling ethanol (approx. 120 ml).

22% **yield**, white solid, **m.p.** = 228.7 °C, **^{1}H-NMR:** (400 MHz, DMSO-d_6) δ = 12.58 (s, 1H), 6.63 (s, 1H), 2.31 (s, 6H). ppm, **^{13}C-NMR:** (101 MHz, DMSO-d_6) δ = 166.8, 160.2 (2C), 115.8, 109.7, 21.9 (2C) ppm, **IR** (KBr) ν = 3503, 3282, 3249, 3064, 3010, 2980, 2857, 2842, 2815, 2621, 2319, 2244, 2202, 2175, 2089, 1838, 1727, 1649, 1610, 1422, 1362, 1323, 1231, 1195, 1165, 1036, 1018, 985 cm^{-1}, **HRMS** (ESI+) *m*/*z*: calcd. for $C_7H_9N_4$ [M+H]$^+$: 149.0827, found: 149.0792.

1-(4,6-Dimethylpyrimidin-2-yl)-3-(4-propoxyphenyl)guanidine

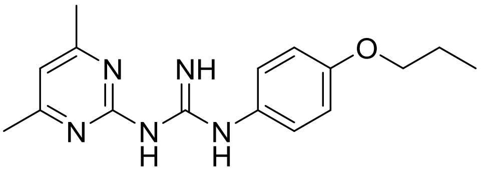

4-Propoxyaniline (307 mg, 2.03 mmol, 1.5 eq.) was added dropwise to a suspension of *N*-(4,6-dimethylpyrimidin-2-yl)cyanamide (200 mg, 1.35 mmol; 1.0 eq.) in anhydrous EtOH (4 ml). Solids were dissolved during addition of aniline. Reaction mixture was heated to reflux. At this temperature reaction mixture was stirred for 72 h and monitored by TLC (eluent: MeOH). Then reaction mixture was cooled to -35 °C and solution of NaOH (10 ml, 10% w/w) was added dropwise. Solids were filtered and washed with Et_2O (4 × 20 ml). Filtrate cake was dissolved in MeOH and purified by column chromatography on silica with MeOH as an eluent.

47% **yield**, white solid, **m.p.** = 191.5 °C, ***R*f =** 0.36 (MeOH, ninhydrine), **^{1}H-NMR:** (400 MHz, Methanol-d_4) δ = 7.21 – 7.08 (m, 2H), 6.92 – 6.83 (m, 2H), 6.61 (s, 1H), 3.89 (t, *J* = 6.5 Hz, 2H), 3.27 (p, *J* = 1.6 Hz, 1H), 2.31 (s, 6H), 1.83 – 1.65 (m, 2H), 1.01 (t, *J* = 7.4 Hz, 3H) ppm, **^{13}C-NMR:** (101 MHz, Methanol-d_4) δ = 168.6 (2C), 165.2, 158.1, 157.3, 133.7, 126.6 (2C), 116.4 (2C), 112.9, 70.9, 23.74 (2C), 23.69, 10.9 ppm, **IR** (KBr) ν = 3312, 3106, 3088, 2959, 2893, 2869, 1631, 1577, 1527, 1509, 1419, 1383, 1344, 1237, 1171, 1117, 1075, 1048, 1024 cm^{-1}, **HRMS** (ESI+) *m*/*z*: calcd. for $C_{16}H_{22}N_5O$ [M+?]$^+$: 300.1824, found: 300.1773.

[1] WO2013/53726, **2013**, *A1*.

NMR spectra

N-(4,6-Dimethylpyrimidin-2-yl)cyanamide

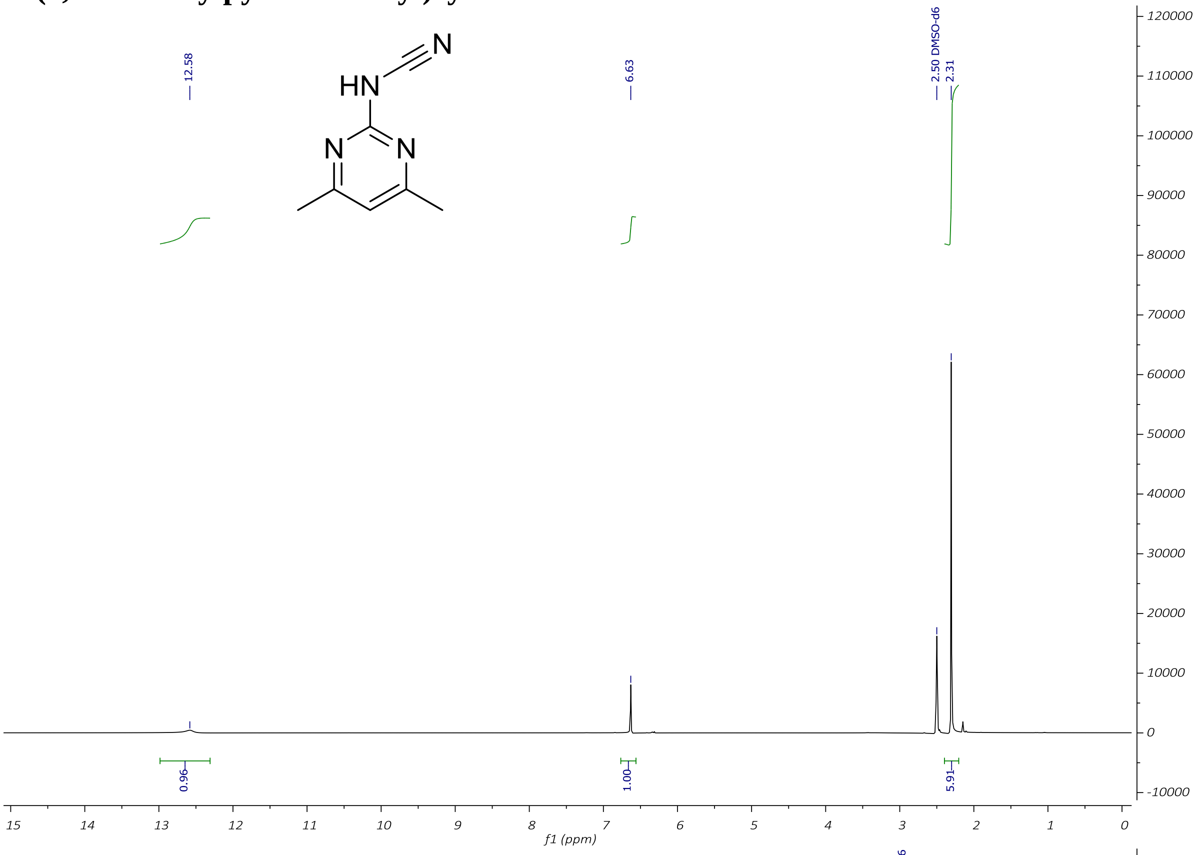

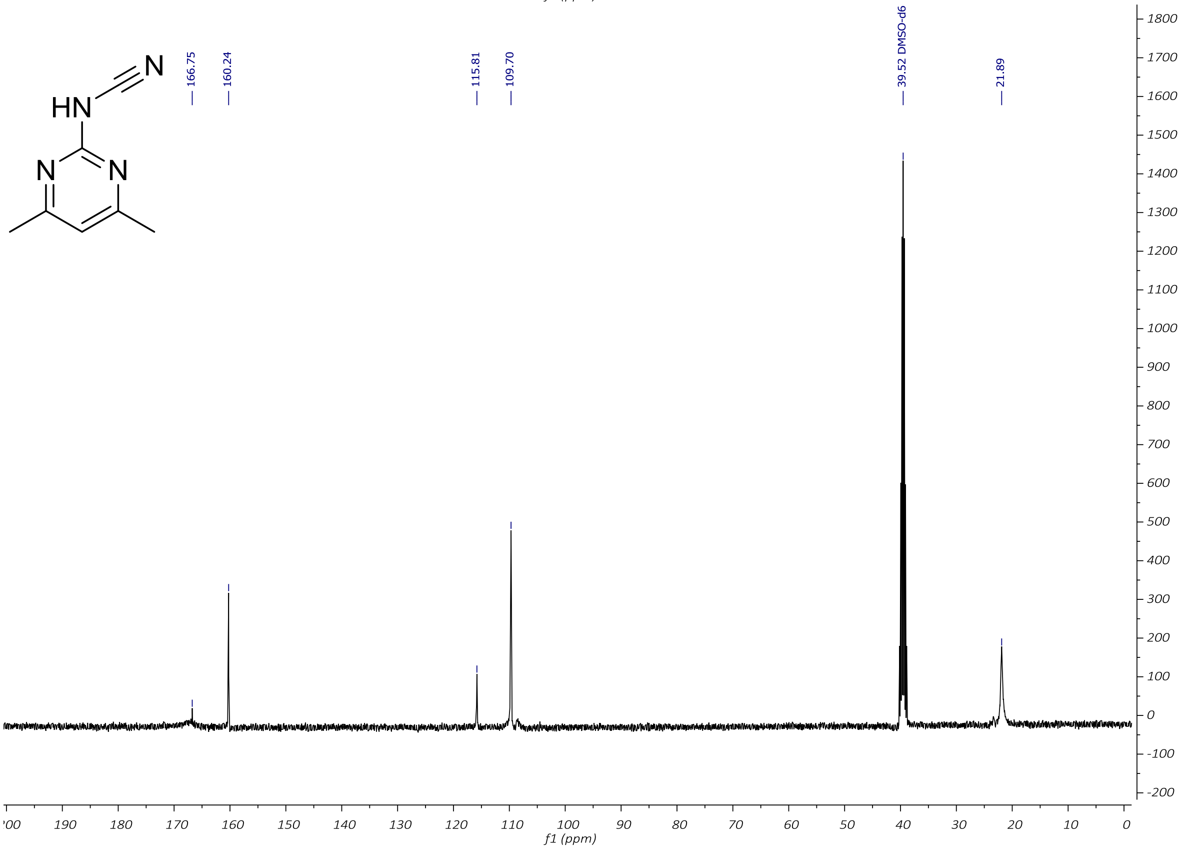

DEPT-135

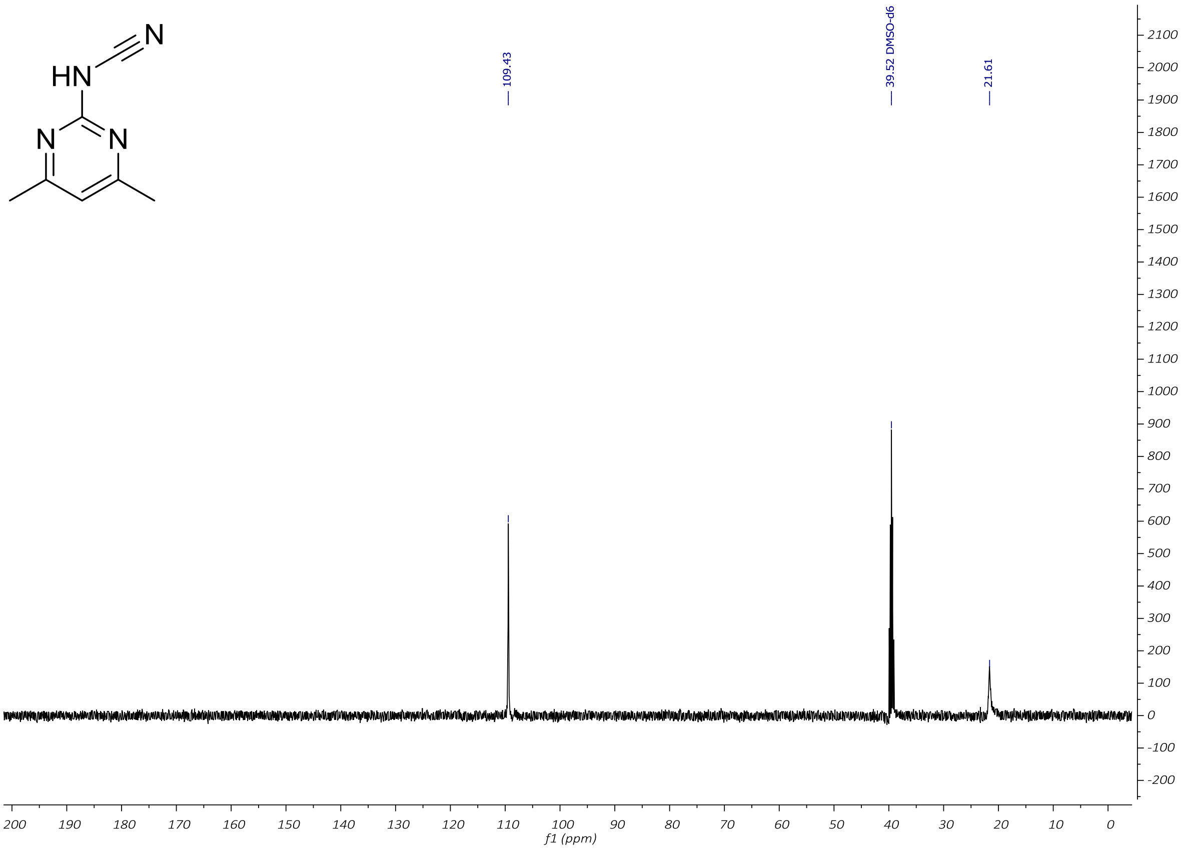

COSY

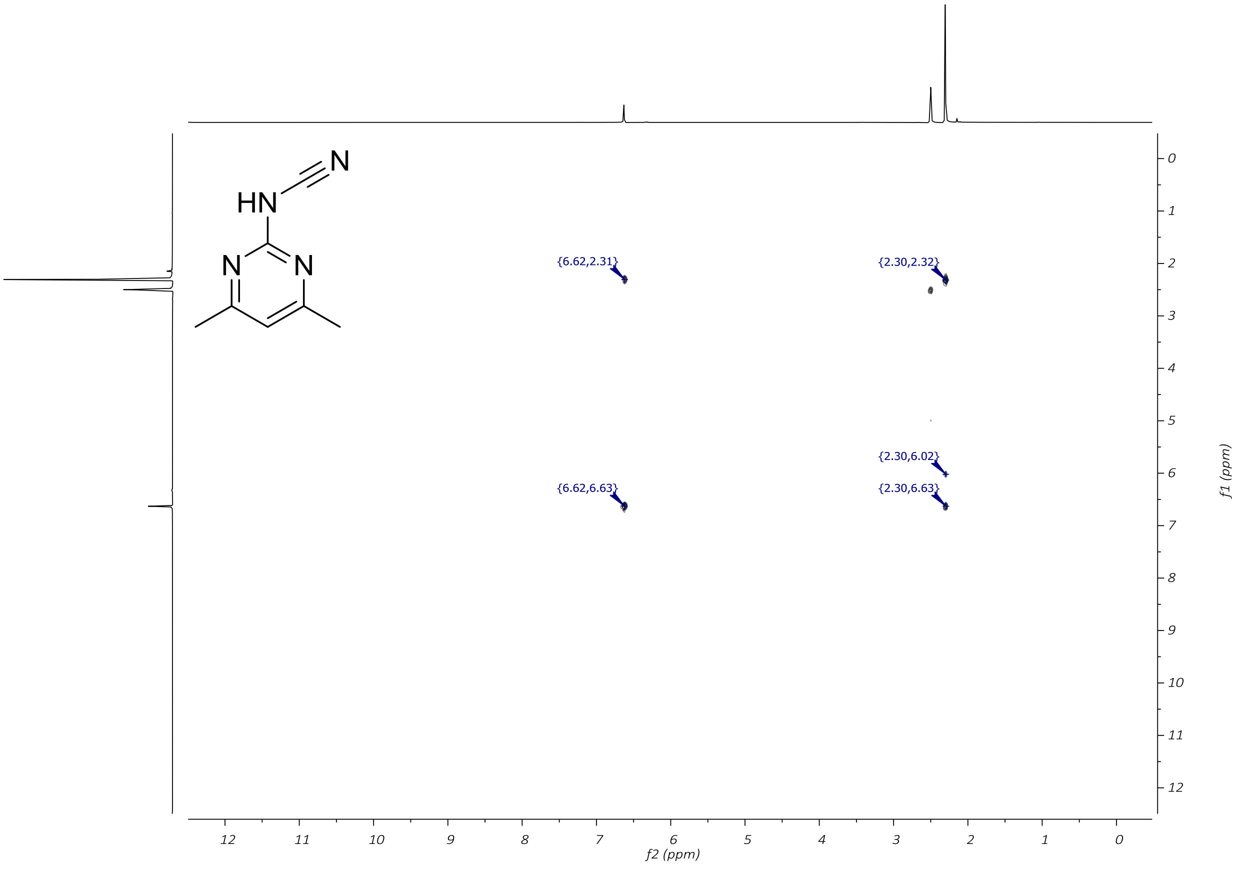

HSQC

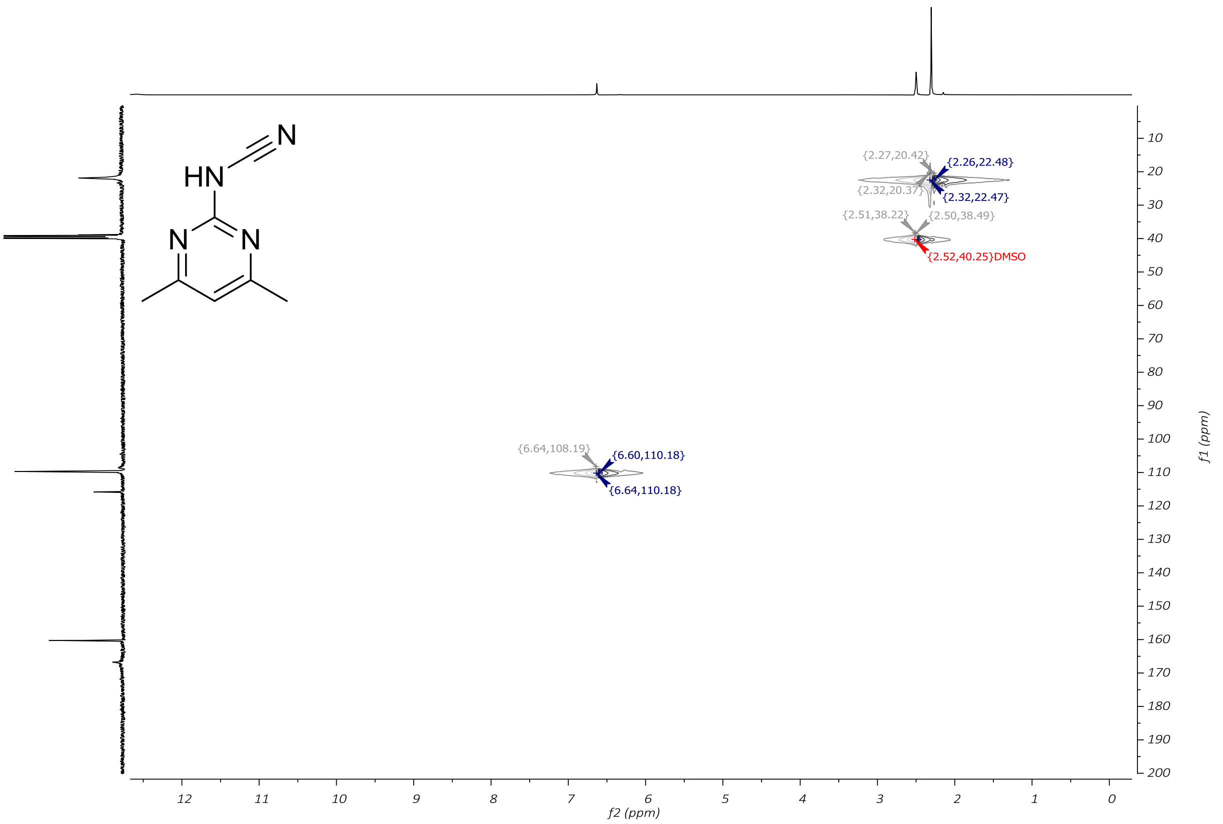

HN
N
N
N
{2.27,20.42}
{2.26,22.48}
{2.32,20.37}
{2.32,22.47}
{2.51,38.22}
{2.50,38.49}
{2.52,40.25}DMSO
{6.64,108.19}
{6.60,110.18}
{6.64,110.18}
f2 (ppm)
f1 (ppm)

HMBC

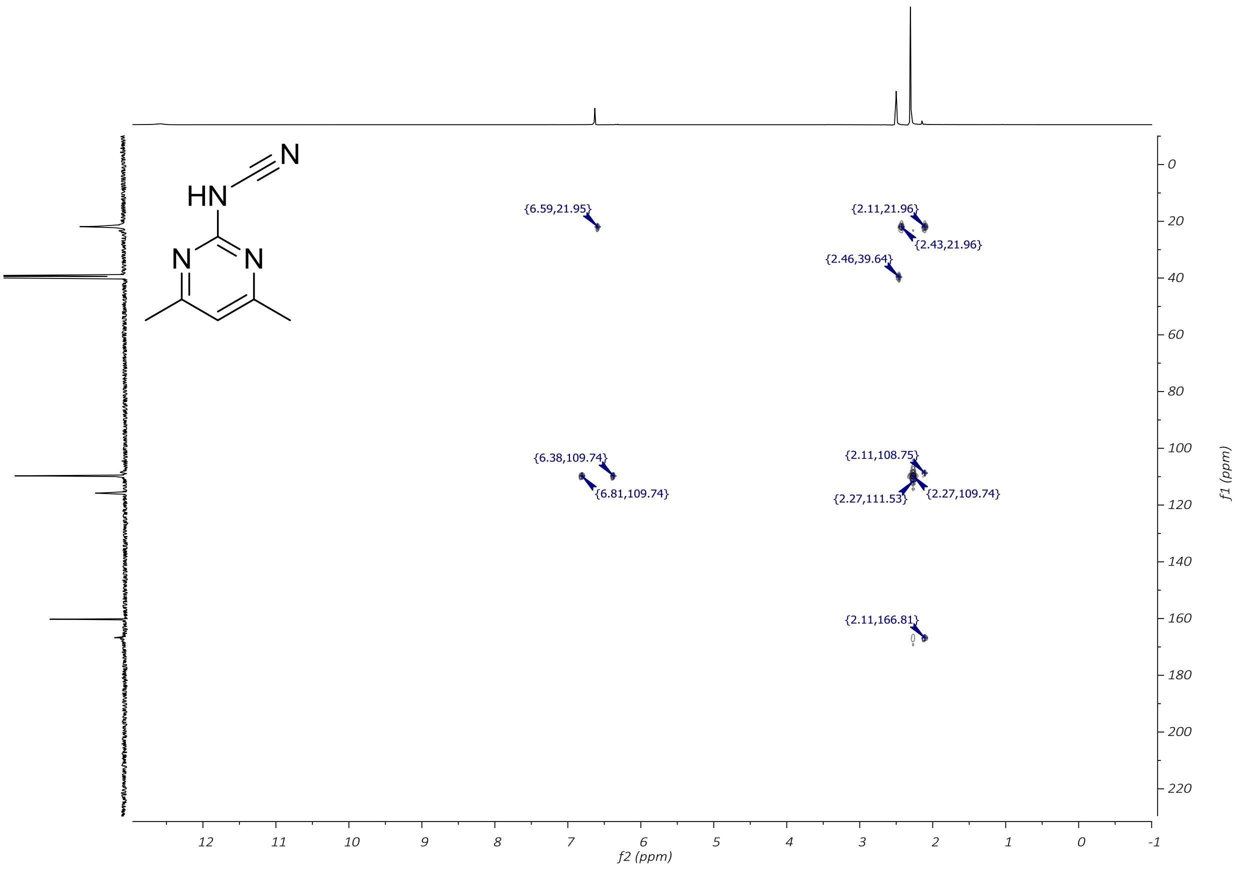

HN
N
N
N
{6.59,21.95}
{2.11,21.96}
{2.43,21.96}
{2.46,39.64}
{6.38,109.74}
{6.81,109.74}
{2.11,108.75}
{2.27,111.53}
{2.27,109.74}
{2.11,166.81}
f2 (ppm)
f1 (ppm)

1-(4,6-Dimethylpyrimidin-2-yl)-3-(4-propoxyphenyl)guanidine

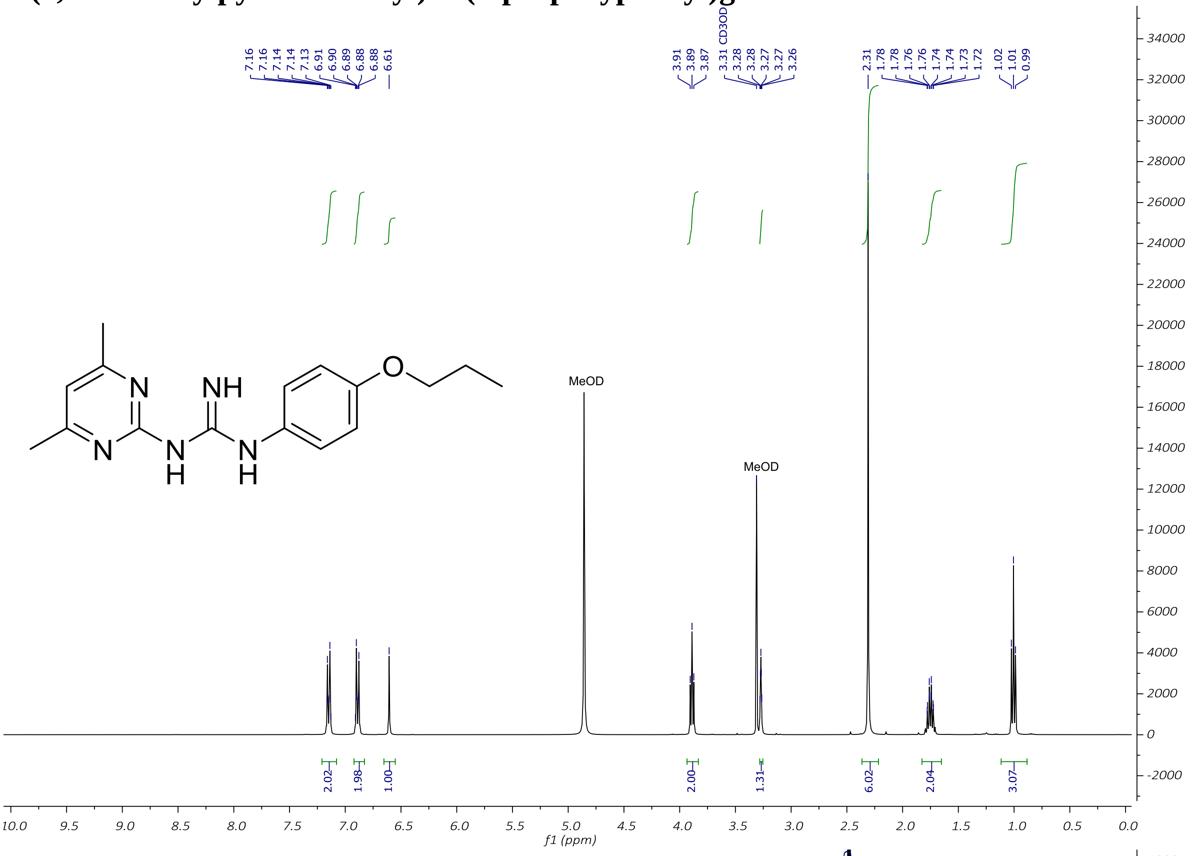

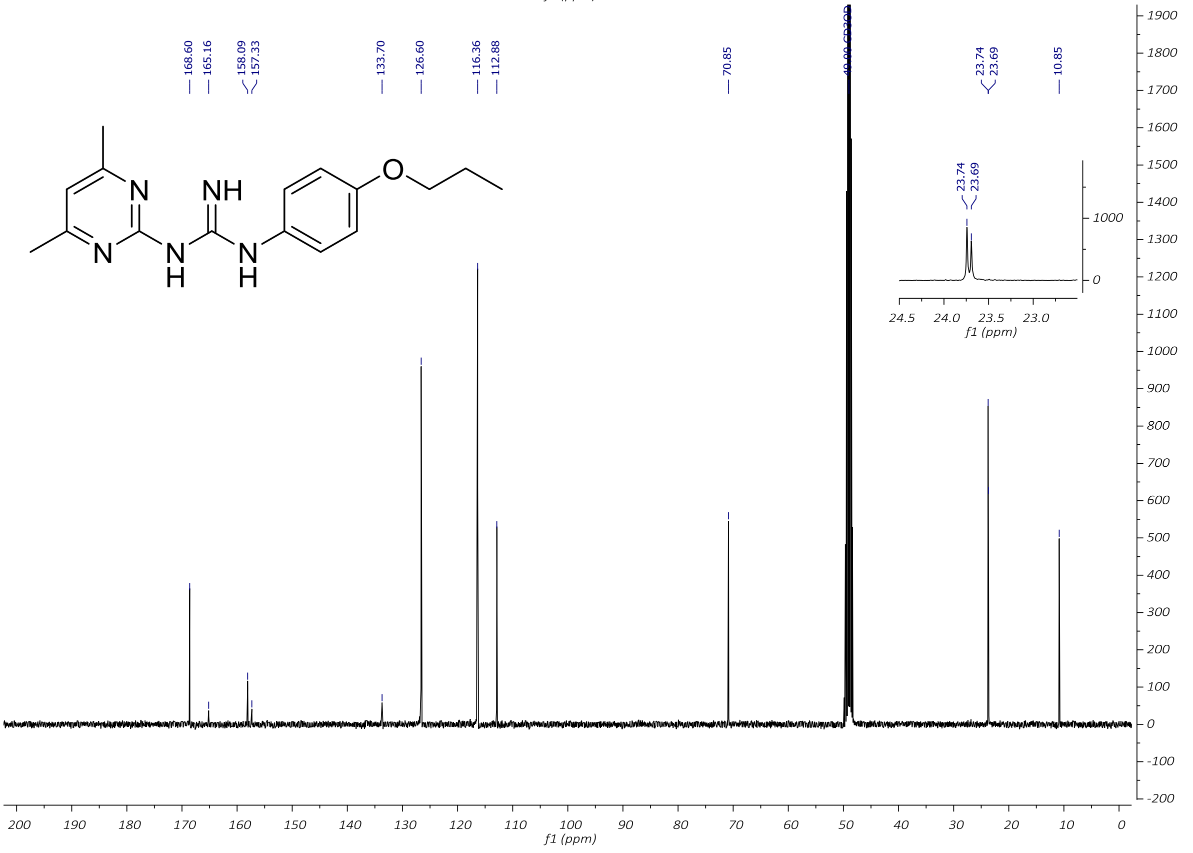

DEPT-135

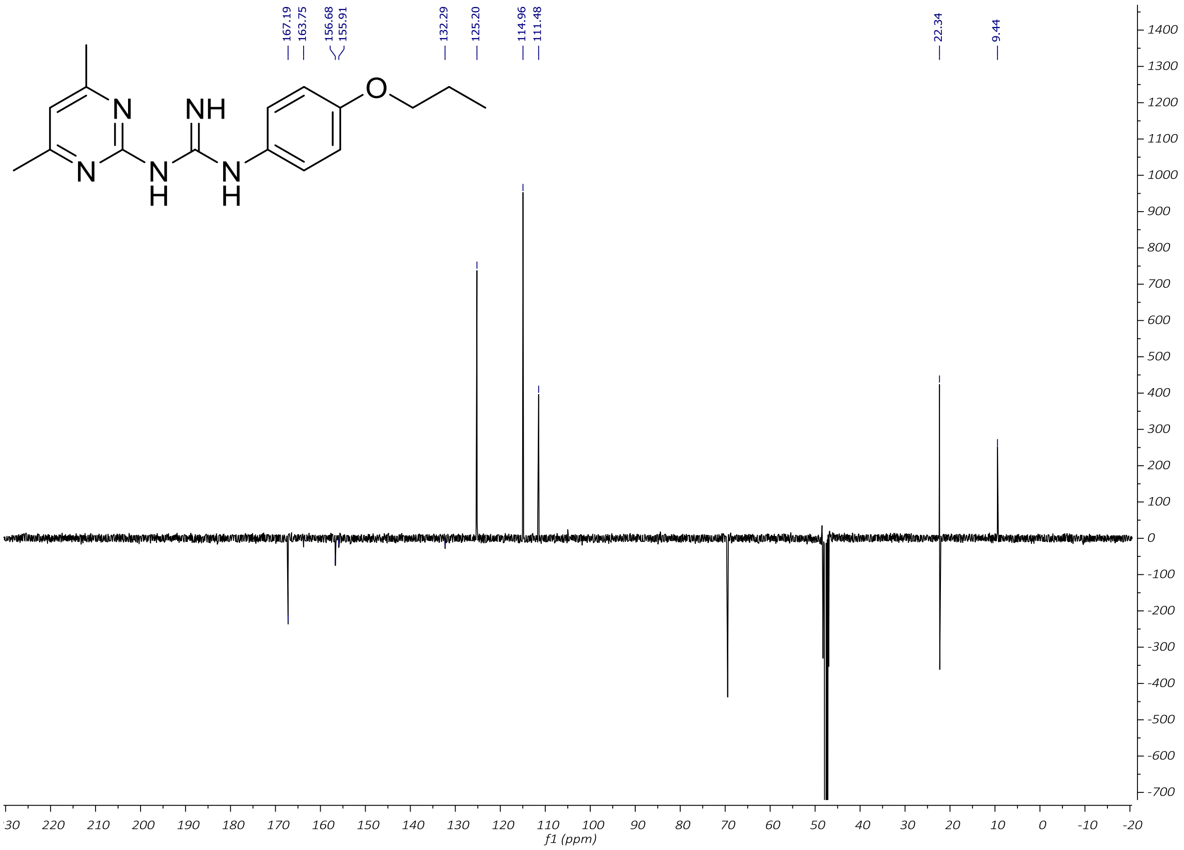

COSY

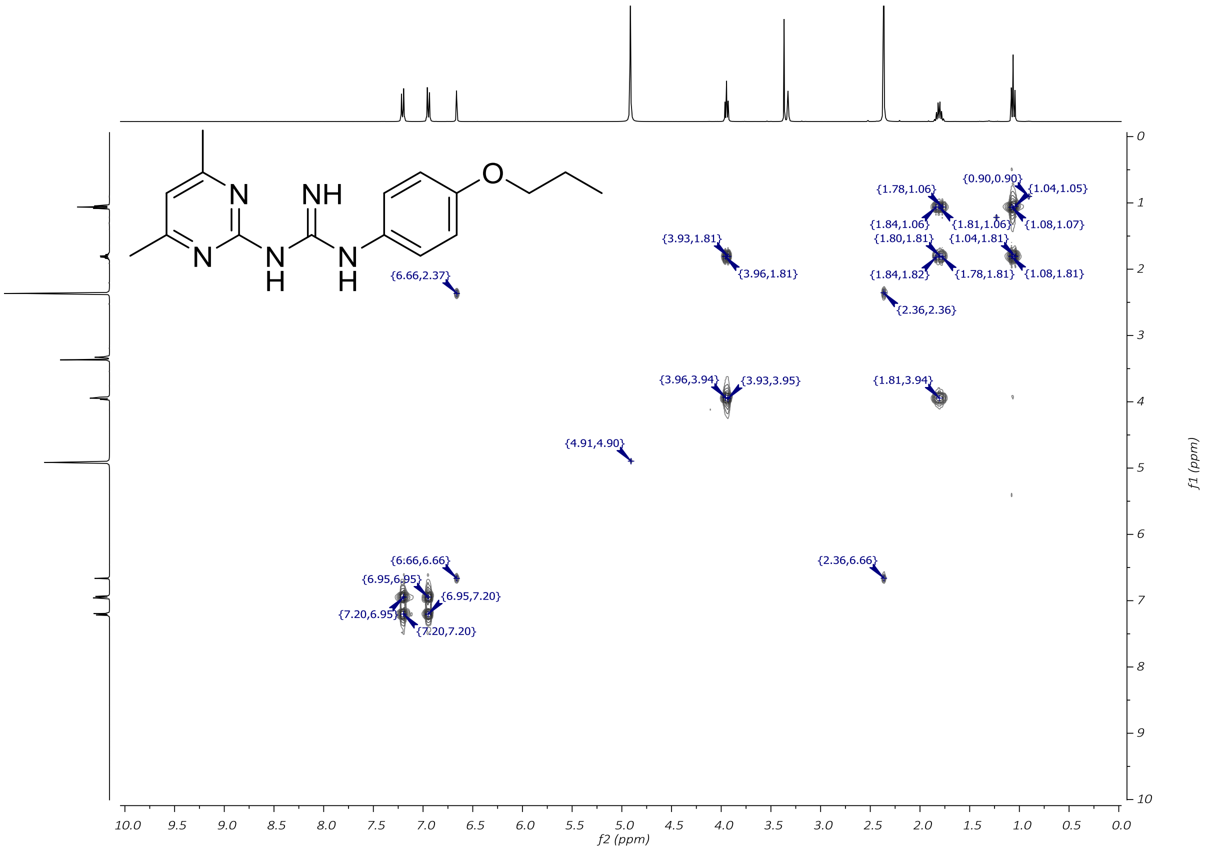

HSQC

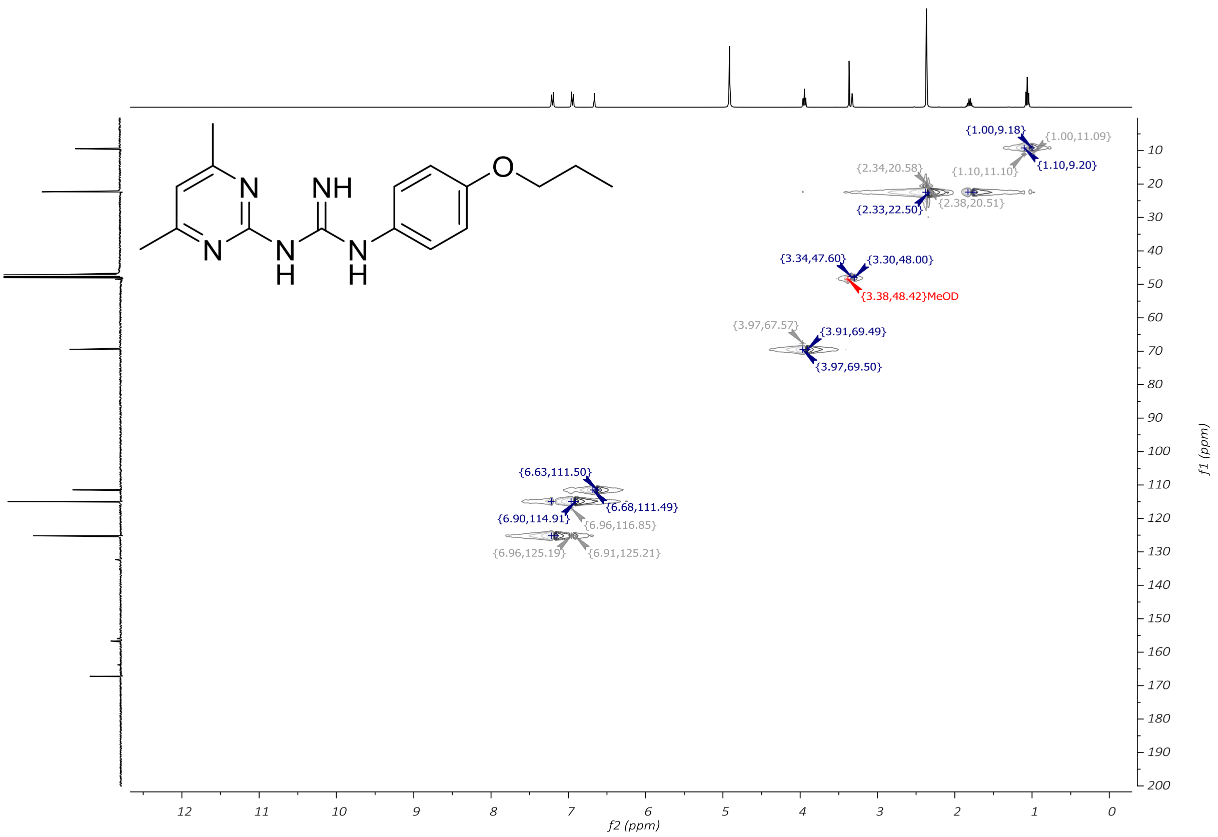

N
NH
O
N
N
H
N
H
{1.00,9.18}
{1.00,11.09}
{1.10,9.20}
{2.34,20.58}
{1.10,11.10}
{2.33,22.50}
{2.38,20.51}
{3.34,47.60}
{3.30,48.00}
{3.38,48.42}MeOD
{3.97,67.57}
{3.91,69.49}
{3.97,69.50}
{6.63,111.50}
{6.68,111.49}
{6.90,114.91}
{6.96,116.85}
{6.96,125.19}
{6.91,125.21}
f2 (ppm)
f1 (ppm)

HMBC

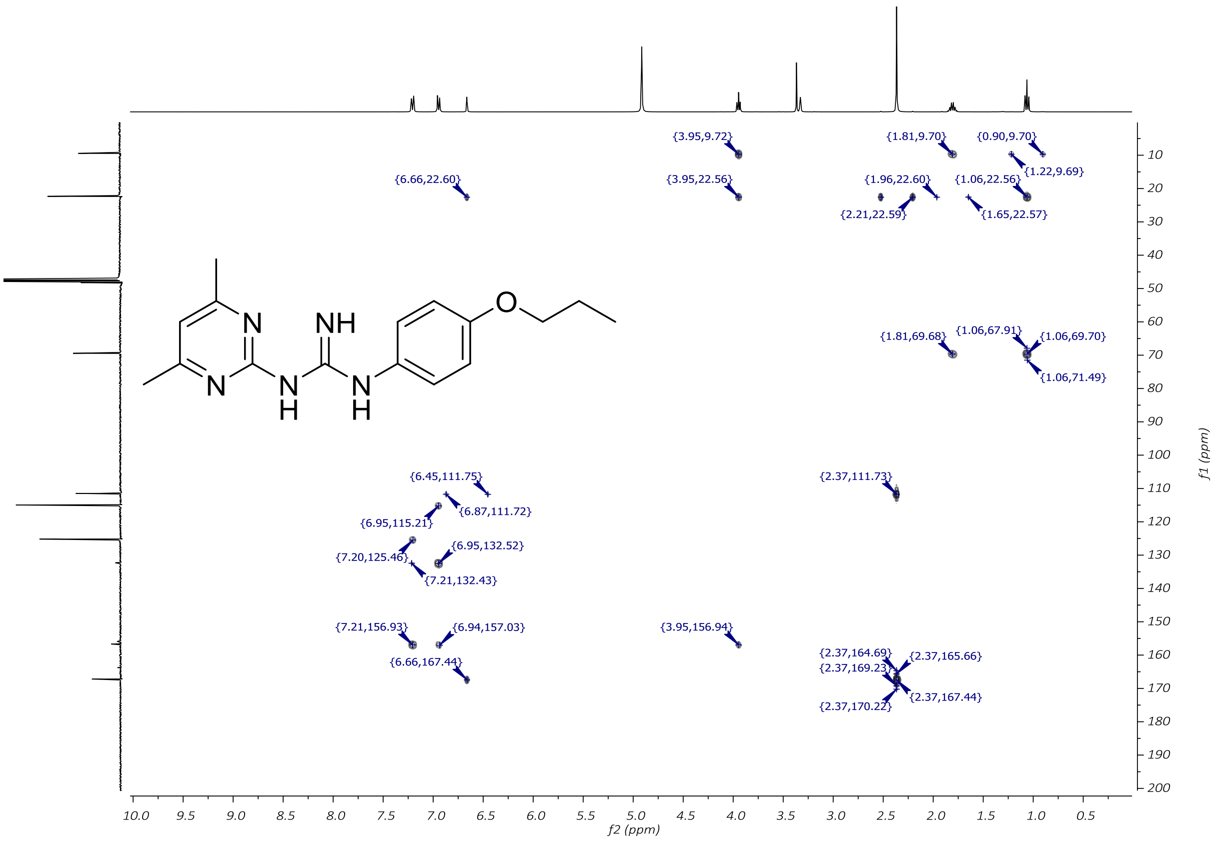

N
NH
O
N
N
H
N
H
{3.95,9.72}
{1.81,9.70}
{0.90,9.70}
{1.22,9.69}
{6.66,22.60}
{3.95,22.56}
{1.96,22.60}
{1.06,22.56}
{2.21,22.59}
{1.65,22.57}
{1.81,69.68}
{1.06,67.91}
{1.06,69.70}
{1.06,71.49}
{6.45,111.75}
{6.87,111.72}
{2.37,111.73}
{6.95,115.21}
{6.95,132.52}
{7.20,125.46}
{7.21,132.43}
{7.21,156.93}
{6.94,157.03}
{3.95,156.94}
{6.66,167.44}
{2.37,164.69}
{2.37,165.66}
{2.37,169.23}
{2.37,167.44}
{2.37,170.22}
f2 (ppm)
f1 (ppm)

Preparation of oxalate salt derived from S9

Corresponding oxalate salt derived from 1-(4,6-dimethylpyrimidin-2-yl)-3-(4-propoxyphenyl)guanidine was prepared according to previously reported procedure.[2]

Oxalate acid dihydrate (27.1 mg; 0.167 mmol; 1.0 eq.) was dissolved in distilled water (1.0 ml). To this solution was added 1-(4,6-dimethylpyrimidin-2-yl)-3-(4-propoxyphenyl) (20.0 mg, 0.167 mmol, 1.0 eq.). Reaction mixture was stirred for 24 h at room temperature. Water was evaporated. Resulting solid was used directly to further studies.

quantitative **yield**, white solid, **^{1}H-NMR:** (400 MHz, DMSO-d_6) δ = 9.18 (s, 2H), 7.38 – 7.22 (m, 2H), 7.18 – 6.94 (m, 3H), 3.96 (t, J = 6.5 Hz, 2H), 2.42 (s, 6H), 1.74 (q, J = 7.0 Hz, 2H), 0.99 (t, J = 7.4 Hz, 3H) ppm, **^{13}C-NMR:** (101 MHz, DMSO-d_6) δ = 168.1 (2C), 165.2, 157.9, 157.2, 154.1 (2C), 127.5 (2C), 126.7, 115.7 (2C), 115.5, 69.3, 23.4 (2C), 22.0, 10.5 ppm.

168.6 (2C), 165.2, 158.1, 157.3, 133.7, 126.6 (2C), 116.4 (2C), 112.9, 70.9, 23.74 (2C), 23.69, 10.9 ppm, **IR** (KBr) ν = 3375, 3294, 3111, 2959, 2881, 1736, 1652, 1613, 1545, 1512, 1428, 1356, 1341, 1299, 1240, 1207, 1180, 1099, 1078, 1054, 1015 cm^{-1}.

[2] Israel, M.; Zoll, E. C.; Muhammad, N.; Modest, E. *J. Med. Chem.* **1973**, *16*, 1-5.

^{1}H-NMR

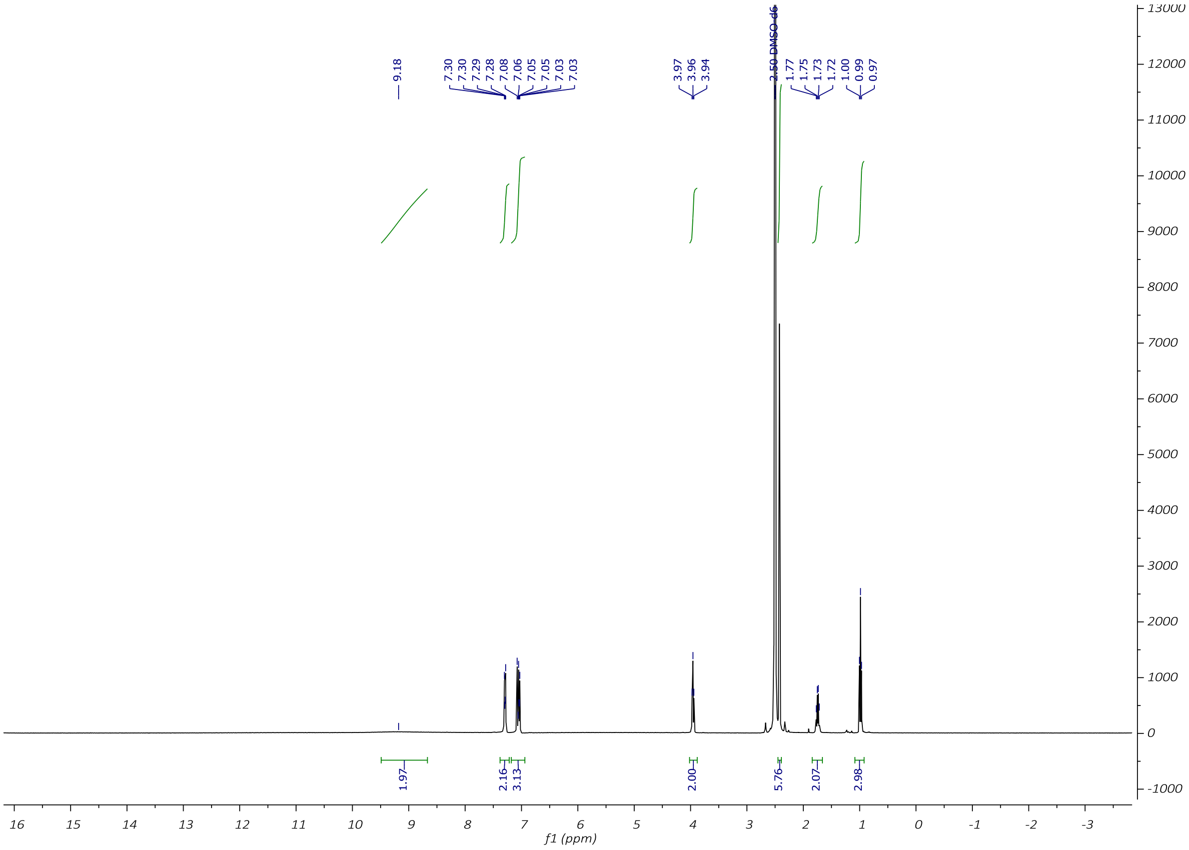

^{13}C-NMR

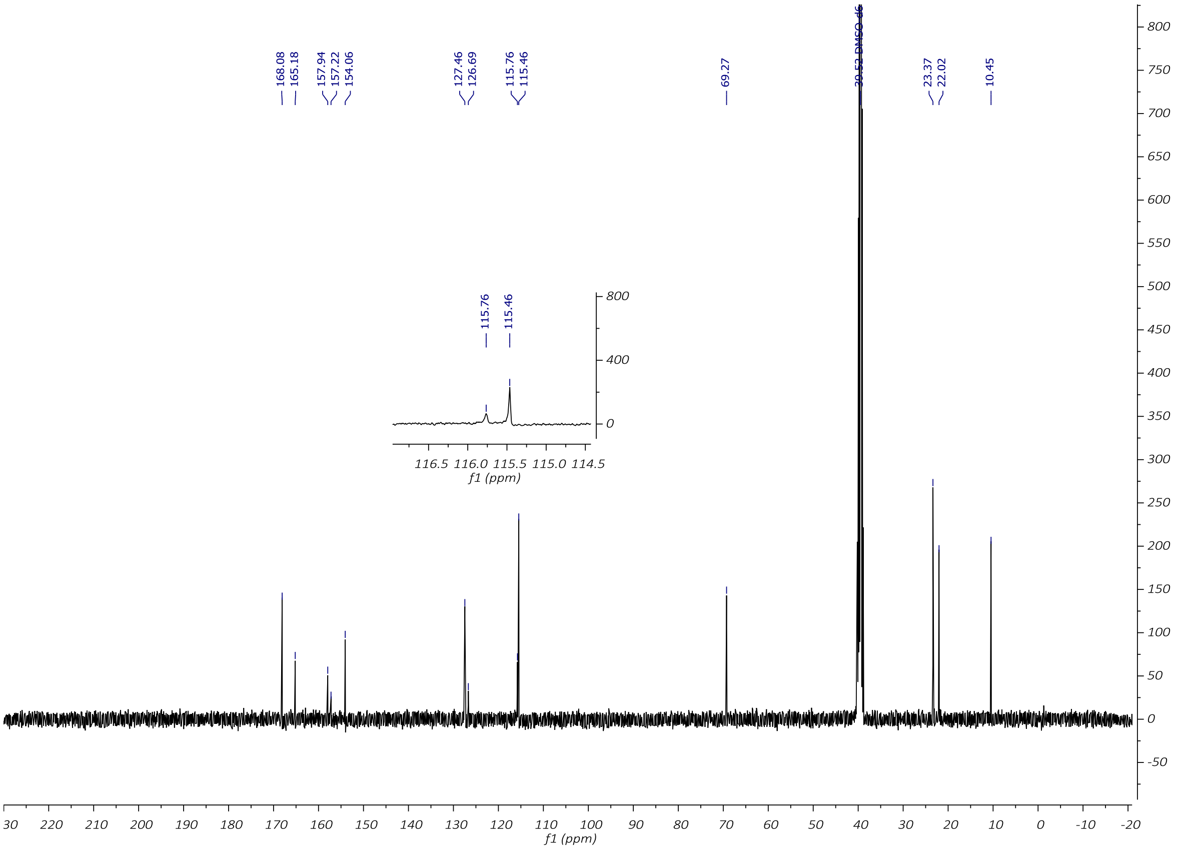

DEPT-135

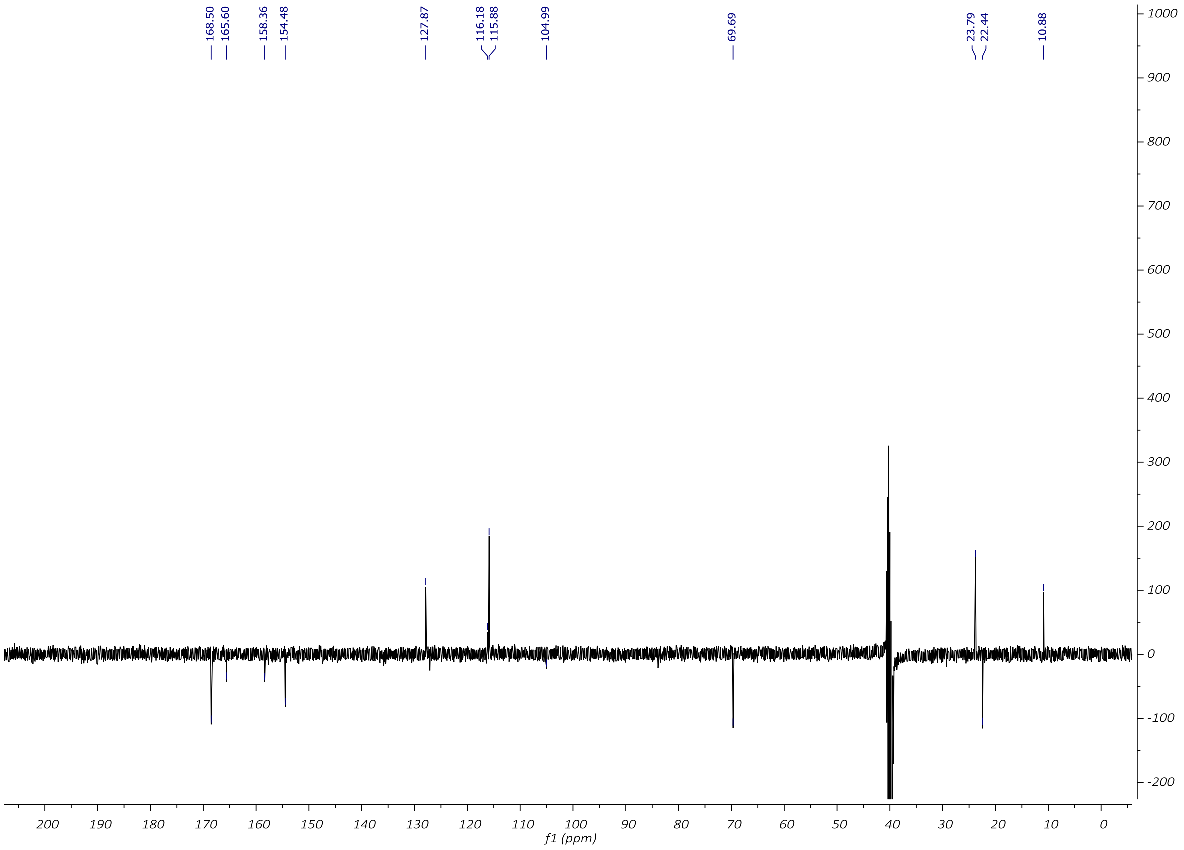

COSY

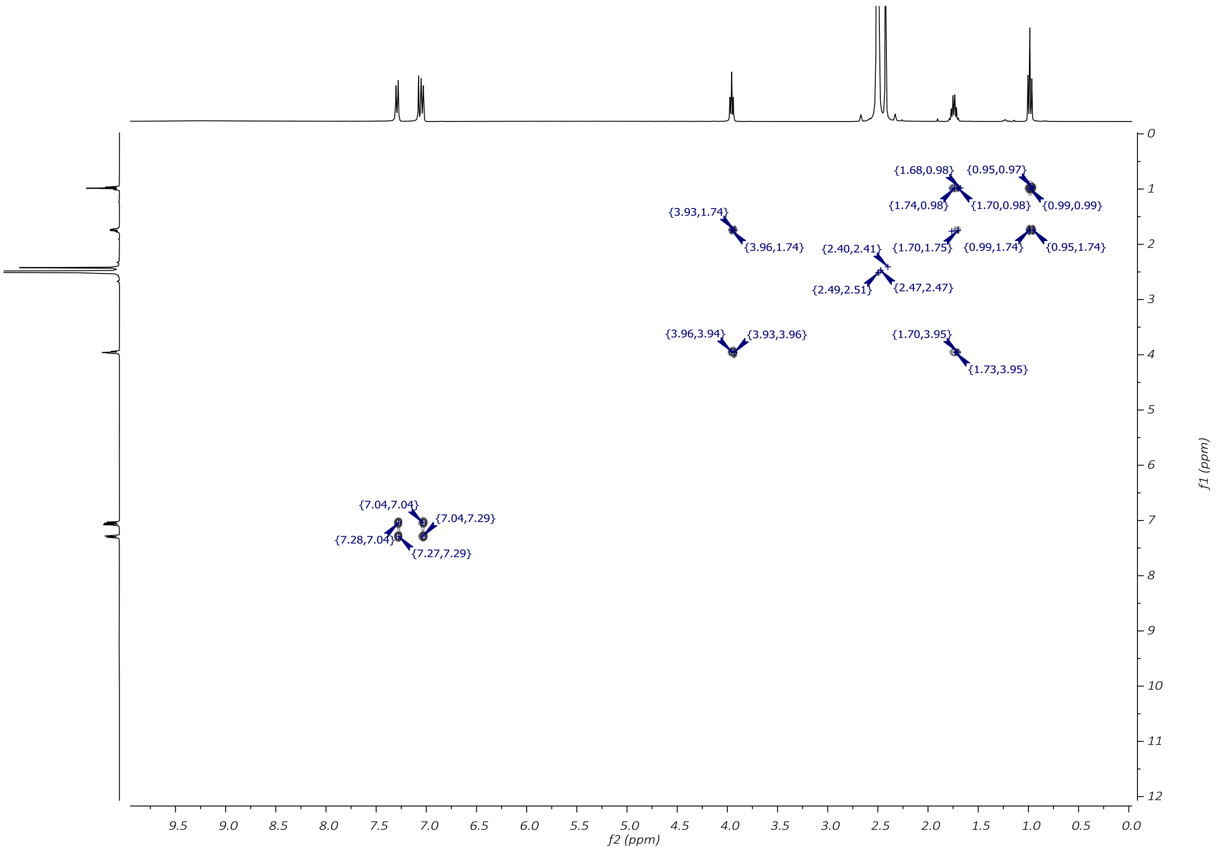

HSQC

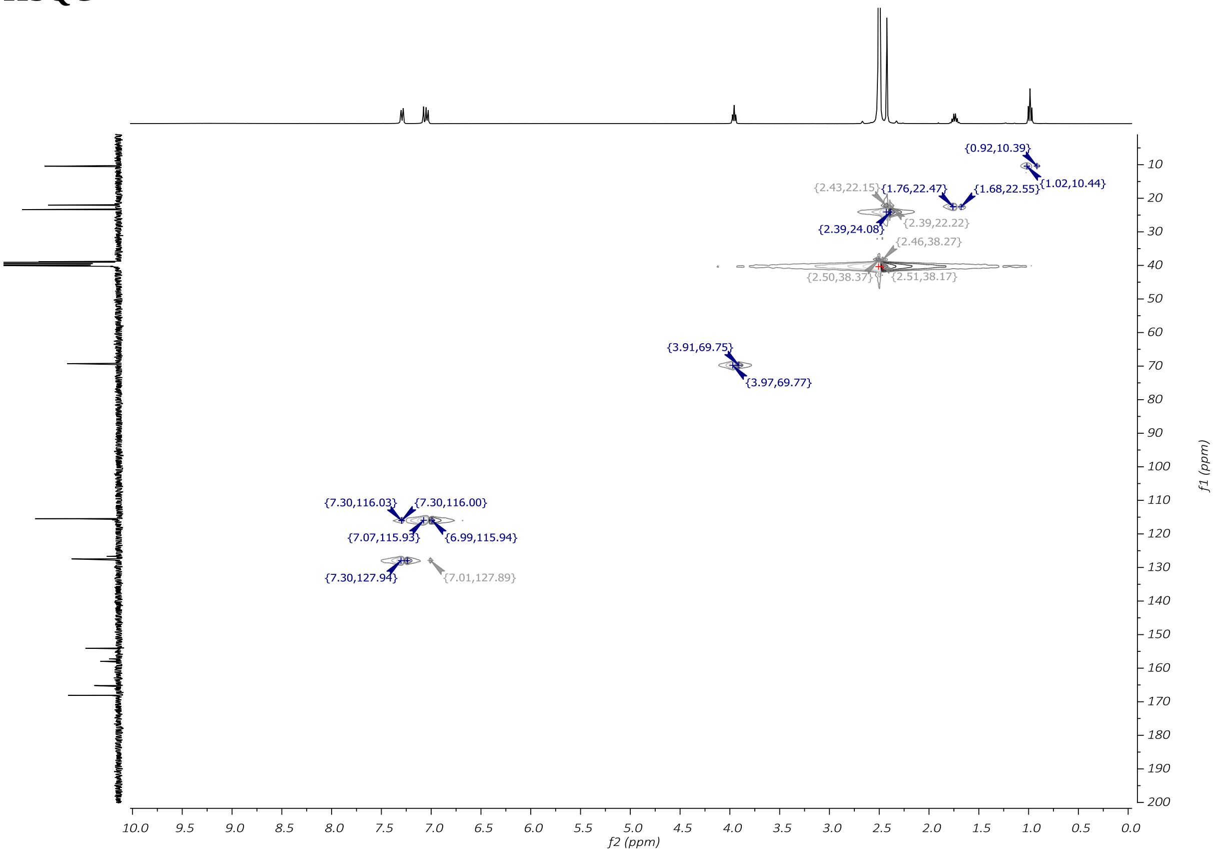
{0.92,10.39}
{1.02,10.44}
{2.43,22.15}
{1.76,22.47}
{1.68,22.55}
{2.39,22.22}
{2.39,24.08}
{2.46,38.27}
{2.50,38.37}
{2.51,38.17}
{3.91,69.75}
{3.97,69.77}
{7.30,116.03}
{7.30,116.00}
{7.07,115.93}
{6.99,115.94}
{7.30,127.94}
{7.01,127.89}
f2 (ppm)
f1 (ppm)

HMBC

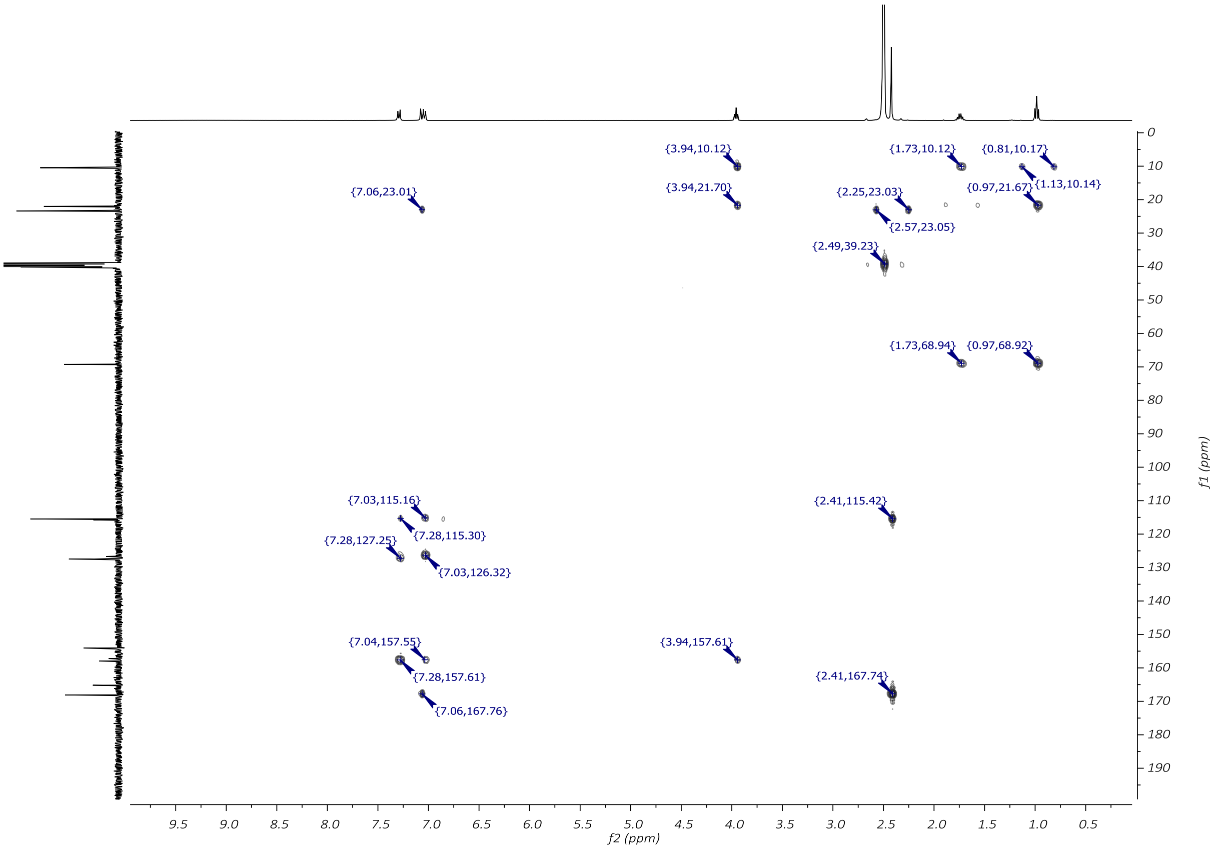
{3.94,10.12}
{1.73,10.12}
{0.81,10.17}
{1.13,10.14}
{7.06,23.01}
{3.94,21.70}
{2.25,23.03}
{0.97,21.67}
{2.57,23.05}
{2.49,39.23}
{1.73,68.94}
{0.97,68.92}
{7.03,115.16}
{7.28,115.30}
{2.41,115.42}
{7.28,127.25}
{7.03,126.32}
{7.04,157.55}
{3.94,157.61}
{7.28,157.61}
{2.41,167.74}
{7.06,167.76}
f2 (ppm)
f1 (ppm)
