# Supplementary material for: Modulating FOXO3 transcriptional activity by small, DBD-binding molecules
Source: eLife. 2019 Dec 4;8:e48876. doi: 10.7554/eLife.48876 (PMC6919977; doi:10.7554/eLife.48876)
Supplement: Supplementary file 2. [file elife-48876-supp2.doc]

| **Key Resources Table** | | | | |
| --- | --- | --- | --- | --- |
| **Reagent type (species) or resource** | **Designation** | **Source or reference** | **Identifiers** | **Additional information** |
| Software, algorithm | LigandScout | (Wolber and Langer 2005) | LigandScout version 3.0 | Pharmacophore modelling |
| Software, algorithm | MOE | (MOE) | MOE version 11.2011 | Molecular Operating Environment |
| Software, algorithm | GOLD | (Jones et al. 1997) | GOLD version 3.1 | Docking |
| Software, algorithm | Drugbank | (Wishart et al. 2008) | Drugbank version 2.5 | Drug data and drug target information base |
| Software, algorithm | Autodock Vina | (Trott and Olson 2010) | Autodock Vina version 1.1.2. | Docking |
| strain (*Escherichia coli*) | BL21(DE3) | Sigma-Aldrich | CMC0016 | Electrocompetent cells |
| cell line (*Homo-sapiens*) | Phoenix-AMPHO | ATCC | ATCC® CRL-3213™ | Packaging cells for amphotropic retroviruses |
| cell line (*Homo-sapiens*) | HEK293T | ATCC | ATCC® CRL-3216™ | Used for packaging of amphotropic lentiviruses |
| cell line (*Homo-sapiens*) | SH-EP/FOXO3 | (Hagenbuchner et al. 2016) | SH-EP RRID:CVCL_0524 | Expression of FOXO(A3)ERtm |
| cell line (*Homo-sapiens*) | NB15/ FOXO3-GFP | (Hagenbuchner et al. 2016) | NB15 RRID:CVCL_A452 | FOXO(A3)ERtm allele and EGFP |
| cell line (*Homo-sapiens*) | SH-EP/ FOXO3-H212R | This paper | SH-EP RRID:CVCL_0524 | Expression of FOXO(A3)ERtm-H212R |
| cell line (*Homo-sapiens*) | SH-EP/ crispCtr | This paper | SH-EP RRID:CVCL_0524 | Carries vector  lentiCRISPR/Cas9 v2 |
| cell line (*Homo-sapiens*) | SH-EP/ crispFOXO3#1 | This paper | SH-EP RRID:CVCL_0524 | Carries lentiCRISPR/Cas9 v2 with gRNA FOXO3#1 |
| cell line (*Homo-sapiens*) | SH-EP/ crispFOXO3#2 | This paper | SH-EP RRID:CVCL_0524 | Carries vector  lentiCRISPR/Cas9 v2 with gRNA FOXO3#2 |
| recombinant DNA reagent | pBabe-puro-HA-FOXO3.A3.ER | (Czymai et al. 2010) |  | Control vector coding for FOXO3(A3)ERtm |
| recombinant DNA reagent | pBabe-puro-HA-FOXO3.A3.ER.H212R | (Czymai et al. 2010) |  | Vector coding for FOXO3(A3)ERtm-H212R mutant |
| recombinant DNA reagent | lentiCRISPR/Cas9 v2 vector | (Sanjana et al. 2014) | Addgene #52961 | Lentiviral CRISPR/Cas9 system |
| recombinant DNA reagent | pLentiCRISPR-FOXO3-1 | This paper |  | Contains gRNA CACCGCCTGCCATATCAGTCAGCCG |
| recombinant DNA reagent | pLentiCRISPR-FOXO3-2 | This paper |  | Contains gRNA CACCGCAGAGTGAGCCGTTTGTCCG |
| recombinant DNA reagent | pGEX-6P-1-FOXO1(159 – 272) | This paper |  | Expression of recombinant human FOXO1-DBD |
| recombinant DNA reagent | pGEX-6P-1-FOXO3(156−269) | This paper |  | Expression of recombinant human FOXO3-DBD |
| recombinant DNA reagent | pET15-FOXO4(82-207) | (Boura et al. 2007) |  | Expression of recombinant human FOXO4-DBD |
| recombinant DNA reagent | pGEX-6P-1-FOXO6(87 - 200) | This paper |  | Expression of recombinant human FOXO6-DBD |
| recombinant DNA reagent | pGEX-6P-1-FOXM1(222-360) | This paper |  | Expression of recombinant human FOXM1-DBD |
| recombinant DNA reagent | pGS c10orf10 Prom wt | (Salcher et al. 2014) |  | Reporter vector containing human DEPP1 promoter |
| recombinant DNA reagent | pGL2-luc-Bim | (Bouillet et al. 2001) |  | Reporter vector containing mouse Bim promoter |
| sequence-based reagent | RT-BIM fwd | (Rupp et al. 2017) |  | qRT-PCR-Primer  AGCACCCATGAGTTGTGACAAATC |
| sequence-based reagent | RT-BIM rev | (Rupp et al. 2017) |  | qRT-PCR-Primer  CGTTAAACTCGTCTCCAATACGC |
| sequence-based reagent | RT-NOXA fwd | (Rupp et al. 2017) |  | qRT-PCR-Primer  AGCAGAGCTGGAAGTCGAGTGTG |
| sequence-based reagent | RT-NOXA rev | (Rupp et al. 2017) |  | qRT-PCR-Primer  TGATGCAGTCAGGTTCCTGAGC |
| sequence-based reagent | RT-SESN3 fwd | (Rupp et al. 2017) |  | qRT-PCR-Primer  GAGGATGTTGACACAACCATGCTG |
| sequence-based reagent | RT-SESN3 rev | (Rupp et al. 2017) |  | qRT-PCR-Primer  CCGCCAGTAACTATCATACATGCG |
| sequence-based reagent | RT-DEPP1 fwd | (Salcher et al. 2014) |  | qRT-PCR-Primer ACTGTCCCTGCTCATCCATTCTC |
| sequence-based reagent | RT-DEPP1 rev | (Salcher et al. 2014) |  | qRT-PCR-Primer AGTCATCCAGGCTAGGAGAGGG |
| sequence-based reagent | RT-GAPDH fwd | (Rupp et al. 2017) |  | qRT-PCR-Primer  TGTTCGTCATGGGTGTGAACC |
| sequence-based reagent | RT-GAPDH rev | (Rupp et al. 2017) |  | qRT-PCR-Primer  GCAGTGATGGCATGGACTGTG |
| sequence-based reagent | ChIP-Bim fwd | (Rupp et al. 2017) |  | ChIP-PCR Primer  GCCGCGCTGGAGTTACAAACTC |
| sequence-based reagent | ChIP-Bim rev | (Rupp et al. 2017) |  | ChIP-PCR Primer TGACTTCCCGGGGTTAGGTAGGAC |
| sequence-based reagent | ChIP-Noxa fwd | (Rupp et al. 2017) |  | ChIP-PCR Primer CATTTCCCTTCCCTGTTACTGCCC |
| sequence-based reagent | ChIP-Noxa rev | (Rupp et al. 2017) |  | ChIP-PCR Primer GGCGGGAGGGGAAGGGTTTA |
| sequence-based reagent | ChIP-SESN3 fwd | (Rupp et al. 2017) |  | ChIP-PCR Primer GACTGAGGTGGATGTGGGTTATTCCTC |
| sequence-based reagent | ChIP-SESN3 rev | (Rupp et al. 2017) |  | ChIP-PCR Primer CATTCCCTTCCCCATCCCTCTC |
| sequence-based reagent | ChIP-DEPP1 fwd | (Salcher et al. 2014) |  | ChIP-PCR Primer CTGCTCCTAGGAGAGACACACC |
| sequence-based reagent | ChIP-DEPP1 rev | (Salcher et al. 2014) |  | ChIP-PCR Primer CTGCTACGTTTGCTGTGCTTAGTGC |
| antibody | Anti-Bim, rabbit mAb (C34C5) | Cell Signaling Technology | #2933 | 1:500 |
| antibody | Anti-FOXO3, rabbit mAb (75D8) | Cell Signaling Technology | #2497 | 1:1000 |
| antibody | Anti-Noxa, mouse mAb, (114C307.1) | Alexis Biochemicals | ALX-804-408 | 1:500 |
| antibody | Anti-SESN3, mouse pAb | Abcam | Ab88454 | 1:500 |
| antibody | Anti-GAPDH, rabbit pAb | Novus Biologicals | NB300-327 | 1:4000 |
| antibody | Anti-DEPP1, rabbit pAb | Sigma (Prestige AB) | HPA037819 | 1:1000 |
| antibody | Anti-α-Tubulin, mouse mAb, (DM1A) | Cell Signaling Technology | #3873 | 1:4000 |
| antibody | Anti-FOXO3, rabbit pAb | Santa Cruz | #sc11351 | ChIP antibody |
| commercial assay or kit | Lipofectamin 2000 | Thermofisher | #11668-019 | Generation of lentiviral particles |
| commercial assay or kit | JetPrime® reagent | Polyplus Transfection | #114-07 | Promoter reporter assays |
| commercial assay or kit | Glutathione Sepharose 4 Fast Flow | Merck | GE17-5132-01 | Purification of GST-tagged proteins |
| commercial assay or kit | Venor^R^ GeM-mycoplasma detection kit | Minerva Biolabs | # 11-1100 | Routine Mycoplasma Testing |
| commercial assay or kit | Luciferase Assay System | Promega GmbH | # E4550 | Promoter reporter assay |
| commercial assay or kit | Maxima™-SYBR-Green-qPCR-Mix | Thermofisher | #K0222 | Quantitative RT PCR |
| commercial assay or kit | Bioprinting Kit | Pelobiotech GmbH | #011-96WK | Magnetic spheroid formation |
| commercial assay or kit | CellTiter-Glow-3D | Promega GmbH | # G9682 | viability of 3D spheroids |
| commercial assay or kit | Magna-ChIP-Kit | Merck | #17-611 | Chromatin immune precipitation |
| commercial assay or kit | RevertAid-H-Minus-cDNA-Synthesis Kit | Thermofisher | #K1632 | RT kit for qRT-PCR |
| commercial assay or kit | ChIP-DNA-Clean-&-Concentrator | Zymo Research | #D5205 | ChIP cleanup |
| commercial assay or kit | MitoTrackerRed CM-H_2_XROS | Thermofisher | #7513 | Reactive oxygene species detection |
